# Dissipative Endoreversible Engine with Given Efficiency

**DOI:** 10.3390/e21111117

**Published:** 2019-11-15

**Authors:** Robin Masser, Karl Heinz Hoffmann

**Affiliations:** Institut für Physik, Technische Universität Chemnitz, 09107 Chemnitz, Germany; robin.masser@physik.tu-chemnitz.de

**Keywords:** endoreversible thermodynamics, non-equilibrium thermodynamics, dissipative engine, irreversibilities, efficiency, entropy production

## Abstract

Endoreversible thermodynamics is a finite time thermodynamics ansatz based on the assumption that reversible or equilibrated subsystems of a system interact via reversible or irreversible energy transfers. This gives a framework where irreversibilities and thus entropy production only occur in interactions, while subsystems (engines, for instance) act as reversible. In order to give an opportunity to incorporate dissipative engines with given efficiencies into an endoreversible model, we build a new dissipative engine setup. To do this, in the first step, we introduce a more general interaction type where energy loss not only results from different intensive quantities between the connected subsystems, which has been the standard in endoreversible thermodynamics up to now, but is also caused by an actual loss of the extensive quantity that is transferred via this interaction. On the one hand, this allows the modeling of leakages and friction losses, for instance, which can be represented as leaky particle or torque transfers. On the other hand, we can use it to build an endoreversible engine setup that is suitable to model engines with given efficiencies or efficiency maps and, among other things, gives an expression for their entropy production rates. By way of example, the modeling of an AC motor and its loss fluxes and entropy production rates are shown.

## 1. Introduction

What is commonly referred to as finite time thermodynamics became increasingly important as early as the 1970s [1]. The modeling of heat engines had to be extended by heat losses and finite rates in order to leave the field of ideal and reversible processes and achieve a better description of real processes occurring in finite time. A particularly interesting and effective approach is endoreversible thermodynamics: The idea is to divide the overall system into reversibly acting subsystems and to connect them via reversible or irreversible interactions. The advantage of this method is that while irreversibilities and hence entropy production can only occur in interactions between the subsystems, the knowledge about equilibrium systems can still be applied to the subsystems.

This simple endoreversible ansatz has proven to be a suitable tool to describe dissipative systems, and since its invention, numerous heat and other energy conversion engines have been modeled [2,3,4,5]. Among them are Carnot cycles in different variations [6,7], in sets of two and more [8,9], or with additional heat leaks [10,11,12], binary distillation processes [13], thermoelectric generators [14,15,16], and solar thermal heat engines [17,18,19,20]. Optimization tasks were carried out, also, for example of the piston trajectories of Otto [21,22], Diesel [23,24], Miller [25,26], Brayton [27], and Stirling [28] cycles, as well as light driven engines [29,30,31]. Recently, it has also been extended to the the study of stochastic heat engines [32,33,34]. Since endoreversible thermodynamics is by no means limited to heat engines, it has also been used to study global wind energy production [35], chemical reactions [36,37,38], and even goods at a market [39], to name a few. The same basic ideas, in particular the optimization of processes, are also used in the context of quantum thermodynamics [40,41,42,43,44,45].

Usually, using the endoreversible formalism, losses or irreversibilities are described as transfers between subsystems with different intensive quantities. For example, in heat engines, the different temperatures of two subsystems between which heat is transferred typically lead to an irreversible process. However, and especially if we leave the field of pure heat engines, irreversibilities can also occur in transfers between subsystems with equal intensive quantities. More generally, the occurring energy losses may not be determined solely by a difference in the named intensive quantities. To take this into account, in this work we, as the first step, introduce a more general description of irreversibilities within the endoreversible formalism.

Further, due to its nature, the endoreversible formalism is especially suitable to study large compound systems consisting of many individual components and with losses occurring within their connections [46,47]. In such cases, the individual components may either have already been studied or may not necessarily be part of the study. For example, engines could be used for which output powers and efficiency are already present as functions of various parameters. These engines must then be incorporated into the endoreversible model considering the given power output and efficiency functions. Therefore, as the second step, using the more general description of irreversibilities introduced, we introduce an engine setup that is suitable to incorporate engines into an endoreversible model for which data such as efficiency and power are already available. This enables reliable predictions to be made about mutual influences between individual components, which are themselves not the object of the investigation.

In the following section, a description of the endoreversible formalism is given, following the review article by Hoffmann et al. [3]. After that, a new and more general interaction type is introduced, which we will refer to as leaky interaction. In the subsequent sections, this new interaction type is used to build an engine model that can be adjusted so that dissipative engines with given efficiencies can easily be modeled and incorporated into an endoreversible description. As an example, we then demonstrate the modeling of an AC motor with given loss functions and conclude our work in the last section.

## 2. General Formalism

The formulation of endoreversible thermodynamics is based on reversible acting subsystems and fluxes of energy or extensive quantities between them. Extensive quantities, short extensities, are quantities that scale with the system size, such as entropy, volume, or particle number. In this work, the extensities of a subsystem *i* are denoted by $X_{i}^{\alpha }$, where α defines the specific type of extensity. Further, for each extensity, there is a conjugate intensive quantity $Y_{i}^{\alpha }$, which does not scale with the system size, such as temperature, pressure, or chemical potential.

If a subsystem *i* has an energy $E_{i}$, according to the Gibbs equation, the change in its energy can be expressed as the sum of changes in extensities times the corresponding intensities:

(1) $$dE_{i}=\sum_{\alpha }Y_{i}^{\alpha }\;dX_{i}^{\alpha }.$$

A change in extensities of the subsystem can, e.g., be caused by an extensity flux $J_{i}^{\alpha }$ out of or into the subsystem. Analogous to Equation (1), the accompanying flux of energy that is carried by an extensity flux is then given by:

(2) $$I_{i}=Y_{i}^{\alpha }J_{i}^{\alpha }.$$

Energy fluxes never occur on their own without a carrying extensity flux. For example, heat can only be transferred when an entropy transfer is involved. However, in endoreversible descriptions, it may happen that the carrying extensity flux is not considered in detail, i.e., it does not matter which type of extensity is carrying the transferred energy. On the other hand, an extensity flux can be transferred without energy being transmitted if the accompanying intensity is zero.

### 2.1. Subsystems

Each subsystem of an endoreversible model has a set of contact points through which it exchanges energy with other subsystems. These contact points are numbered by *k*. Since the transferred energy is carried by an extensity, the corresponding intensity $Y_{i,k}^{\alpha }$ is assigned to that contact point.

Furthermore, the subsystems are divided into three functionally distinct categories, namely reservoirs, engines, and chemical reactors. In the case of reservoirs, the associated intensity at a contact point is the intensive quantity of the reservoir. Reservoirs act as storages for energy and extensities and represent equilibrated systems. We can further distinguish between finite and infinite reservoirs.

When a reservoir is finite, its state is given by its energy as a function of its extensive quantities:

(3) $$E_{i}=E_{i}\left(X_{i}^{\alpha }\right),$$

and its intensive quantities can be calculated as the partial derivatives of the energy:

(4) $$Y_{i}^{\alpha }=\frac{\partial E_{i}\left(X_{i}^{\alpha }\right)}{\partial X_{i}^{\alpha }}.$$

Thus, the state of the subsystem varies with the variation of its extensities, which in general can be expressed as:

(5) $${\dot{X}}_{i}^{\alpha }=\sum_{k}J_{i,k}^{\alpha }+\Sigma_{i}^{\alpha },$$

where $J_{i,k}^{\alpha }$ are fluxes into or out of the subsystem at contact points *k* and $\Sigma_{i}^{\alpha }$ is an extensity generation or destruction term. The latter might be necessary for non-conserved extensive quantities, e.g., when chemical reactions occur within the subsystem.

If we consider an infinite reservoir, the intensities remain constant, independent of the fluxes into or out of the reservoir. It represents an infinite large system with fixed intensive quantities, and its state is thus described by them. Typical examples for such infinite systems are heat baths with constant temperature or reservoirs with constant pressure.

Engines, on the other hand, can neither store energy nor extensities. They are meant to convert energy between different incoming and outgoing extensity fluxes. Since engines work reversibly, no extensity is generated or destroyed within them. Thus, the balance equations for extensities and energy for the engine *i* can be written as:

(6) $$\begin{array}{cc} 0 & =\sum_{k}J_{i,k}^{\alpha }\;\mathrm{for}\;\mathrm{all}\;\alpha \;\mathrm{and} \end{array}$$

(7) $$\begin{array}{cc} 0 & =\sum_{k}I_{i,k}=\sum_{\alpha ,k}Y_{i,k}^{\alpha }J_{i,k}^{\alpha }, \end{array}$$

respectively, where *k* denotes the $k^{\mathrm{th}}$ contact of the engine.

Unlike in reservoirs, the intensities associated with the contact points of an engine are freely selectable for each contact point. This is an important feature that allows a reversible connection between reservoirs with different states, as needed, e.g., to model a perfect regenerator in a Stirling engine.

The chemical reactor, which is the third type of endoreversible subsystems and which was introduced by Wagner [48,49], is slightly different from the endoreversible engine. However, this subsystem type, as well as chemical reactions in principal are not considered and hence not explained in detail in this work.

The endoreversible formalism is by no means limited to the classical thermodynamic quantities of entropy, volume, and particle number. Among others, mechanical quantities such as momentum and angular momentum or electrical quantities such as charge can easily be incorporated, as well. Setting the properties of a subsystem *i* still occurs via the definition of $E_{i}\left(X_{i}^{\alpha }\right)$, which can include, e.g., translational energy, rotational energy, and electric potential energy. Since Equation (4) applies, the corresponding intensities velocity, angular velocity, or electric potential, respectively, can be derived.

### 2.2. Reversible and Irreversible Interactions

Interactions in endoreversible thermodynamics are used to describe how energy and extensities are exchanged between the subsystems. An interaction is always assigned to one or more contact points of each of the subsystems that are linked via this interaction. It is thus characterized by the set of contact points that belong to the interaction and by the specific type of extensity carrying the exchanged energy.

Since interactions are not meant to produce or destroy energy, also, energy is balanced over all contact points linked by an interaction. The same holds for all conserved extensities. Whether an interaction is reversible or irreversible, i.e., generates entropy or not, depends on its definition. First, in the case of a reversible interaction, the intensities at the contact points connected via this interaction obey:

(8) $$Y_{i,k}^{\alpha }=Y_{j,l}^{\alpha },$$

where *k* and *l* denote connected contact points of the subsystems *i* and *j*, respectively. In this case, the transfer rates between the subsystems can become infinitely large in order to ensure an instantaneous equilibration of the intensities.

Second, in case of an irreversible interaction, a transport law is used to define either the flux of the interaction’s extensity or the flux of energy. This transport law may be a function of intensities, extensities (in the case of reservoirs), and additional parameters. A typical example is Newton’s heat transfer law, which is a function of the intensities and the temperatures of the connected subsystems, with an additional parameter, the heat transfer coefficient, defining the energy that is exchanged between the subsystems.

**Figure 1:**
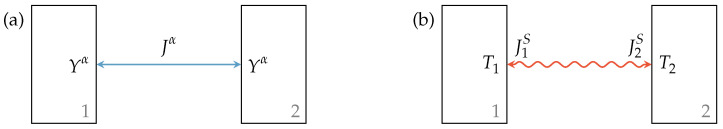
(**a**) Reversible interaction with arbitrary extensity and (**b**) irreversible interaction with heat transfer between two subsystems.

While in the case of a reversible interaction, as shown in Figure 1a, the amounts of both the extensity and the energy fluxes are equal at all contact points:

(9) $$\begin{array}{cc} J_{1}^{\alpha } & =J^{\alpha }=-J_{2}^{\alpha }, \end{array}$$

(10) $$\begin{array}{cc} I_{1} & =Y^{\alpha }J^{\alpha }=-I_{2}, \end{array}$$

in the case of an irreversible heat transfer, displayed in Figure 1b, only energy conservation holds:

(11) $$\begin{array}{cc} J_{1}^{S} & \neq -J_{2}^{S}, \end{array}$$

(12) $$\begin{array}{cc} I_{1} & =K\left(T_{2}-T_{1}\right)=-I_{2}, \end{array}$$

where *K* is the heat transfer coefficient and $T_{1}$ and $T_{2}$ are the temperatures of Reservoirs 1 and 2, respectively. With the entropy flux $J_{1}^{S}=I_{1}/T_{1}$ entering or leaving Subsystem 1, the entropy flux into or out of Reservoir 2 is given by:

(13) $$J_{2}^{S}=\frac{I_{2}}{T_{2}}=-J_{1}^{S}\frac{T_{1}}{T_{2}}=-J_{1}^{S}+\underset{\sigma }{\underset{︸}{J_{1}^{S}\frac{T_{2}-T_{1}}{T_{2}}}},$$

where σ is the entropy generation rate due to the difference in intensities of the subsystems.

**Figure 2:**
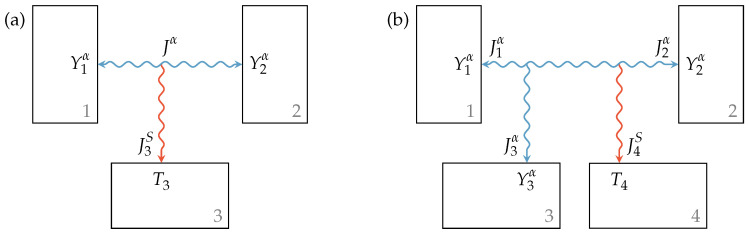
(**a**) Irreversible interaction between reservoirs with different intensities and (**b**) lossy interaction between reservoirs with different intensities where a loss extensity transfer occurs towards a third reservoir. In both cases, the generated entropy is transferred to another additional reservoir.

For the Newtonian heat transfer, the generated entropy is typically transferred to the contact point with lower temperature. However, if contact points with extensity type α other than entropy are involved in an irreversible interaction, as shown in Figure 2a, an additional entropy reservoir is needed to receive the generated entropy. In this case, the extensity flux is conserved:

(14) $$J_{1}^{\alpha }=J^{\alpha }=-J_{2}^{\alpha }$$

but energy escapes into a reservoir with temperature $T_{3}$ carried by an entropy flux:

(15) $$J_{3}^{S}=\frac{J^{\alpha }\left(Y_{2}^{\alpha }-Y_{1}^{\alpha }\right)}{T_{3}}=\frac{I_{3}}{T_{3}}=\sigma ,$$

where σ, again, is the entropy generation rate. This entropy generation rate is positive for all physically feasible definitions of the transfer law used, i.e., for an extensity transfer from the point of higher intensity to the point of lower intensity.

## 3. Leaky Interaction

Now, we can imagine a third and more general option to define an interaction between two subsystems in endoreversible thermodynamics, which we will refer to as leaky interaction. We set this interaction so that Reservoirs 1 and 2 may or may not have the same intensity, but the transfer of extensities or the transfer of energy is “leaky” and thus lossy to some extent. Hence, the fluxes entering or leaving those reservoirs do not balance, and a reservoir receiving the excess extensity flux:

(16) $$-J_{3}^{\alpha }=J_{1}^{\alpha }+J_{2}^{\alpha }$$

is needed, as shown in Figure 2b. Here, the energy balance is not yet fulfilled:

(17) $$0\neq Y_{1}^{\alpha }J_{1}^{\alpha }+Y_{2}^{\alpha }J_{2}^{\alpha }+Y_{3}^{\alpha }J_{3}^{\alpha }=\Delta I,$$

and the generated entropy has to be transferred to, in general, another Subsystem 4 with temperature $T_{4}$ by the entropy flux:

(18) $$\begin{array}{c} J_{4}^{S}=-\frac{\Delta I}{T_{4}}=\frac{J_{2}^{\alpha }\left(Y_{1}^{\alpha }-Y_{2}^{\alpha }\right)}{T_{4}}+\frac{J_{3}^{\alpha }\left(Y_{1}^{\alpha }-Y_{3}^{\alpha }\right)}{T_{4}}=\sigma . \end{array}$$

As it applies to Equation (15), the entropy generation rate σ given in the above equation is positive for physically feasible extensity fluxes, and in these cases, the second law of thermodynamics applies to this interaction.

Such an interaction may find application especially in mechanical systems. Consider two components with angular momentum, e.g., two rotating flywheels, which are connected by a solid shaft and hence have equal angular velocities. We assume that there is no torsion within the shaft, so an reversible interaction transferring angular momentum between two reservoirs with the same angular velocities would describe this setup. However, if the shaft is supported by a bearing causing friction loss, the above described leaky interaction can be used. The lost angular momentum is transferred to the bearing, which can be described by an infinite reservoir with zero angular velocity, since the bearing is typically mounted to a housing or to the floor. Then, the dissipated energy flows together with the generated entropy flux into a heat bath with ambient temperature.

We want to emphasize here that for the described leaky interaction, an additional law defining the loss flux of the specific extensity ($J_{3}^{\alpha }$ in the figures above) is needed. In the case of the bearing with friction considered above, this could be a function of the intensities of the reservoirs involved. Of course, dependencies on other extensities or additional parameters are generally possible, as well. Here, an important feature is that the resulting energy loss occurs regardless of the direction of the main energy exchange between two subsystems.

## 4. Engine Setup with Leaky Interaction

**Figure 3:**
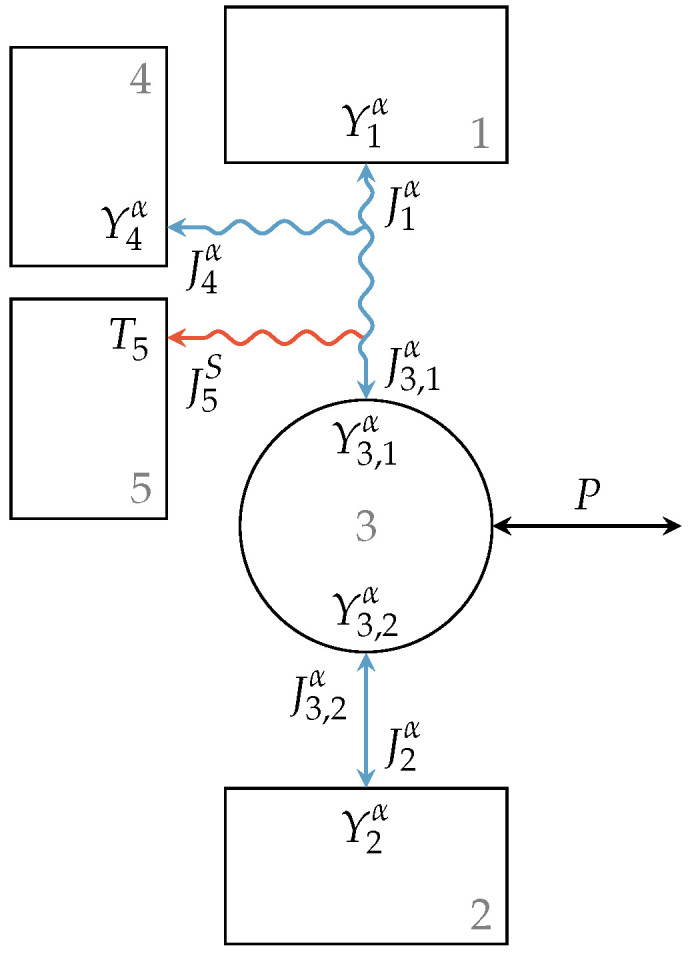
Engine setup with lossy extensity transfer. The upper reservoir is connected to Engine 3 via a lossy interaction with loss extensity and entropy flux towards Reservoirs 4 and 5, respectively, while the lower reservoir is reversibly connected to the engine, which further has an unspecified power output.

In the following, we will consider an engine setup with a leaky interaction representing losses of extensity, which could be more in line with the intuitive modeling of endoreversible non-heat engines. In Figure 3, this setup is shown. A leaky interaction as it is described in the previous section connects Reservoir 1 with Engine 3. The lost extensity α of this interaction is transferred to Reservoir 4 and generated entropy to Reservoir 5. Engine 3 is further reversibly connected to Reservoir 2. The intensive quantities of Reservoirs 1, 2, 4, and 5 are $Y_{1}^{\alpha }$, $Y_{2}^{\alpha }$, $Y_{4}^{\alpha }$, and $T_{5}$, respectively, while the intensities at the engines’ contact points are $Y_{3,1}^{\alpha }$ and $Y_{3,2}^{\alpha }$, respectively.

In general extensities are conserved within the lossy interaction, as well as within the engine so that the equations:

(19) $$\begin{array}{cc} J_{3,1}^{\alpha } & =-J_{1}^{\alpha }-J_{4}^{\alpha }, \end{array}$$

(20) $$\begin{array}{cc} J_{3,2}^{\alpha } & =-J_{3,1}^{\alpha } \end{array}$$

hold, respectively. From the reversibility of the interaction between Engine 3 and Reservoir 2, we can further conclude:

(21) $$\begin{array}{cc} J_{3,2}^{\alpha } & =-J_{2}^{\alpha }, \end{array}$$

(22) $$\begin{array}{cc} Y_{3,2}^{\alpha } & =Y_{2}^{\alpha }. \end{array}$$

In the case of the irreversible interaction between Reservoir 1 and Engine 3, the intensities $Y_{1}^{\alpha }$ and $Y_{3,1}^{\alpha }$ are not equal in general. A phenomenological relation can be used to describe the resulting dissipation. This applies also to the dissipation due to the loss extensity flux $J_{4}^{\alpha }$.

If we now assume, e.g., $Y_{1}^{\alpha }\geq Y_{3,1}^{\alpha }\geq Y_{2}^{\alpha }$, this engine setup can operate in a motor mode where an extensity transfer from Reservoir 1 to the engine leads to a power output *P* that can be written as:

(23) $$\begin{array}{cc} P & =-J_{3,1}^{\alpha }Y_{3,1}^{\alpha }-J_{3,2}^{\alpha }Y_{3,2}^{\alpha } \end{array}$$

(24) $$\begin{array}{cc} \; & =-J_{3,1}^{\alpha }\left(Y_{3,1}^{\alpha }-Y_{3,2}^{\alpha }\right) \end{array}$$

(25) $$\begin{array}{cc} \; & =\left(J_{1}^{\alpha }+J_{4}^{\alpha }\right)\left(Y_{3,1}^{\alpha }-Y_{3,2}^{\alpha }\right). \end{array}$$

Note that $J_{1}^{\alpha }$ and $J_{4}^{\alpha }$ typically would have opposite signs. Here, the transfer of useful energy is the output power *P*, whereas the input energy transfer to the engine is $I_{1}=J_{1}^{\alpha }Y_{1}^{\alpha }$. If we further assume that the energy transfer to Reservoir 2 is of no use, the efficiency η of this engine can be expressed as:

(26) $$\eta =\frac{\left(J_{1}^{\alpha }+J_{4}^{\alpha }\right)\left(Y_{3,1}^{\alpha }-Y_{3,2}^{\alpha }\right)}{J_{1}^{\alpha }Y_{1}^{\alpha }}.$$

## 5. Dissipative Engine with Given Efficiency

We might encounter a modeling task in which we want to incorporate real engines with given efficiency data, or data of any other performance measure, into an endoreversible model. These data typically have been recorded in test runs and describe the actual losses occurring within the engine. The typical approach would be to approximate the measured data using an endoreversible model of the engine itself where simple phenomenological relations are used for all internally occurring losses. For a better approximation, an arbitrarily extensive model with complicated material laws is used. However, the resulting model is always just an approximation of the measured data and, even with considerable effort, can only be incorporated in calculations under the loss of information.

The previously introduced engine setup with leaky extensity transfer now offers a new option. It gives us a set of coupled balance equations that can easily be solved for the desired variables. We then simply incorporate the given efficiencies, or other performance measures of the engine, as external parameters to obtain an engine model without losing the information or complexity of the recorded data.

For example, if extensity α is transferred at $Y_{1}^{\alpha }=Y_{3,1}^{\alpha }$, we can easily define the loss flux $J_{4}^{\alpha }$ so that the overall efficiency of the engine setup equals a given measured efficiency $\eta_{G}$. Assuming the motor mode discussed above, where energy is transferred from extensity α to an unspecified power output by transferring it from a high intensity reservoir to a low intensity reservoir, in analogy to Equation (26), the given efficiency of the engine setup can be expressed as:

(27) $$\eta_{G}=\frac{\left(J_{1}^{\alpha }+J_{4}^{\alpha }\right)\left(Y_{1}^{\alpha }-Y_{2}^{\alpha }\right)}{J_{1}^{\alpha }Y_{1}^{\alpha }},$$

where we used $Y_{3,1}^{\alpha }=Y_{1}^{\alpha }$ and $Y_{3,2}^{\alpha }=Y_{2}^{\alpha }$. Solving Equation (27) for $J_{4}^{\alpha }$, we obtain:

(28) $$J_{4}^{\alpha }=J_{1}^{\alpha }\left(\eta_{G}\frac{Y_{1}^{\alpha }}{Y_{1}^{\alpha }-Y_{2}^{\alpha }}-1\right)$$

as the desired loss flux for the given efficiency $\eta_{G}$.

A given performance measure might also apply to the opposite direction of action, where the power input causes a transfer of extensity α from a lower intensity reservoir to a higher intensity reservoir as for instance in a heat pump. In this case, where we can also assume $Y_{1}^{\alpha }\geq Y_{2}^{\alpha }$, the transfer of useful energy from the engine may be:

(29) $$P_{\mathrm{out}}=J_{1}^{\alpha }Y_{1}^{\alpha }.$$

The energy input transfer to the engine may be given by:

(30) $$P_{\mathrm{in}}=P=\left(J_{1}^{\alpha }+J_{4}^{\alpha }\right)\left(Y_{1}^{\alpha }-Y_{2}^{\alpha }\right)$$

so that energy transferred from Reservoir 2 is assumed to be for free. Combining the equations above, the given performance measure $\lambda_{G}$, which can be larger than one and therefore would be called a coefficient of performance, can be written as:

(31) $$\lambda_{G}=\frac{P_{\mathrm{out}}}{P_{\mathrm{in}}}=\frac{J_{1}^{\alpha }}{J_{1}^{\alpha }+J_{4}^{\alpha }}\frac{Y_{1}^{\alpha }}{Y_{1}^{\alpha }-Y_{2}^{\alpha }}.$$

The corresponding loss flux $J_{4}^{\alpha }$ according to the given performance measure can then be calculated as:

(32) $$J_{4}^{\alpha }=J_{1}^{\alpha }\left(\frac{1}{\lambda_{G}}\frac{Y_{1}^{\alpha }}{Y_{1}^{\alpha }-Y_{2}^{\alpha }}-1\right).$$

Of course, the given performance measure is not restricted to be constant, but can be a function of intensities $\left\{Y_{i}^{\alpha }\right\}$, extensities $\left\{X_{j}^{\alpha }\right\}$, fluxes $\left\{J_{k}^{\alpha }\right\}$, or additional parameters $\left\{z_{l}\right\}$:

(33) $$\lambda_{G}=\lambda_{G}\left(\left\{Y_{i}^{\alpha }\right\},\left\{X_{j}^{\alpha }\right\},\left\{J_{k}^{\alpha }\right\},\left\{z_{l}\right\}\right),$$

and can be easily incorporated as tabulated values, for instance. The generated entropy of the overall engine setup for both conversion directions yields:

(34) $$\sigma =J_{5}^{S}=J_{4}^{\alpha }\frac{Y_{1}^{\alpha }-Y_{4}^{\alpha }}{T_{5}}$$

and is transferred to Reservoir 5 for which in most cases, an infinite entropy reservoir with ambient temperature is suitable.

**Figure 4:**
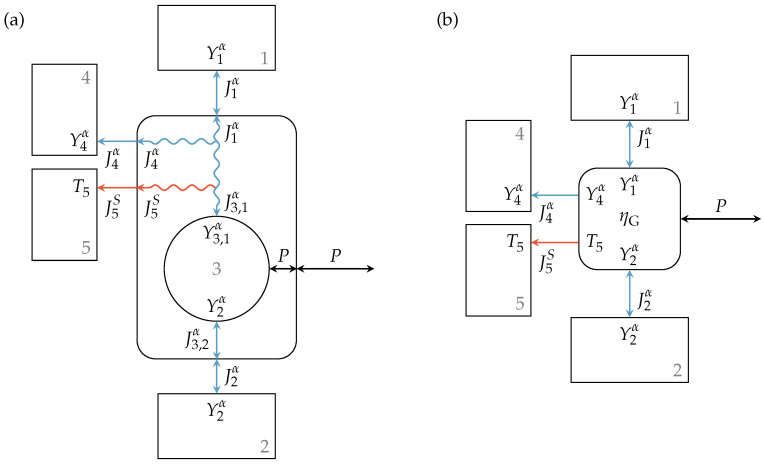
Simplification of (**a**) the engine with lossy extensity transfer to (**b**) a black box dissipative engine with given efficiency. The resulting black box model (rectangle with rounded corners) operates between Reservoirs 1 and 2 with efficiency $\eta_{G}$, while the loss extensity is reversibly transferred to Reservoir 4 and the generated entropy to Reservoir 5.

In principle, this also allows a kind of black box view of this engine setup as a dissipative engine with given efficiency as illustrated in Figure 4, where the new engine type is illustrated as a rectangle with rounded corners. Using the above equations for loss fluxes and entropy generation, non-heat engines with arbitrary efficiency functions can be incorporated into an endoreversible description of large composite systems with ease. In particular, for engineering applications where many different components are assembled and synergy effects or thermodynamic effects have to be investigated, this black box model can be very beneficial. It is easy to handle while it precisely maps the relevant performance parameters of the engines.

## 6. Full Model of a Dissipative Engine

**Figure 5:**
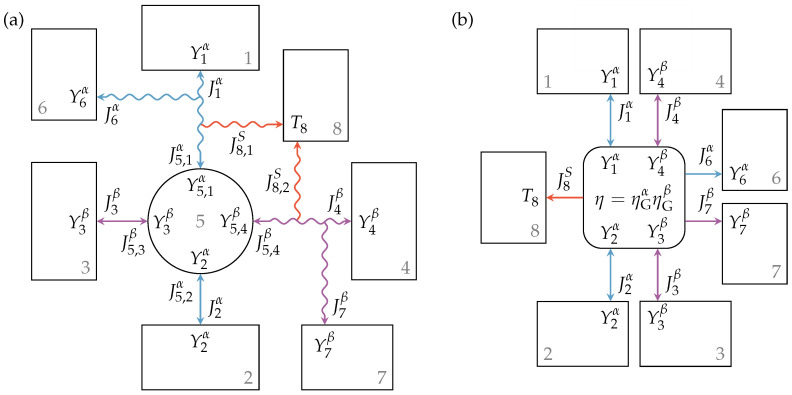
Engine setup with lossy extensity transfer for both extensities α and β (**a**) and the corresponding black box model with given efficiencies (**b**). The engine operates between Reservoirs 1 and 2 and Reservoirs 3 and 4 with efficiency η, while loss extensity transfers of extensities α and β occur towards Reservoirs 6 and 7, respectively, and the generated entropy is transferred to Reservoir 8.

The above description can also be applied to engine setups with two specified extensity types where energy is converted from extensity α to extensity β. The endoreversible model for this engine setup is shown in Figure 5a. Here, the flux of extensity α from Reservoir 1 to Reservoir 2 drives a flux of extensity β from Reservoir 3 to Reservoir 4, or vice versa. Reservoirs 2 and 3 are reversibly connected to Engine 5, while Reservoirs 1 and 4 are connected via a lossy extensity transfer. The latter are those reservoirs with higher intensities, hence $Y_{1}^{\alpha }\geq Y_{2}^{\alpha }$ and $Y_{4}^{\beta }\geq Y_{3}^{\beta }$. Loss extensity fluxes enter Reservoirs 6 and 7, and the generated entropy is transferred to Heat bath 8 with temperature $T_{8}$. As illustrated, the intensities at the contact points of the engine are equal to those of the linked reservoirs. From the extensity balance equation of the reversible interactions and Engine 5, we obtain:

(35) $$\begin{array}{cc} J_{2}^{\alpha } & =-J_{5,2}^{\alpha }=J_{5,1}^{\alpha }, \end{array}$$

(36) $$\begin{array}{cc} J_{3}^{\beta } & =-J_{5,3}^{\beta }=J_{5,4}^{\beta }, \end{array}$$

where $J_{5,1}^{\alpha }$, $J_{5,2}^{\alpha }$, $J_{5,3}^{\beta }$, and $J_{5,4}^{\beta }$ are the fluxes entering or leaving Engine 5 at the contact points towards Reservoirs 1 to 4, respectively, and $J_{2}^{\alpha }$ and $J_{3}^{\beta }$ are the fluxes into or out of Reservoirs 2 and 3, respectively. The relations of the extensity fluxes of the lossy interactions are given by:

(37) $$\begin{array}{cc} 0 & =J_{1}^{\alpha }+J_{5,1}^{\alpha }+J_{6}^{\alpha }, \end{array}$$

(38) $$\begin{array}{cc} 0 & =J_{4}^{\beta }+J_{5,4}^{\beta }+J_{7}^{\beta }. \end{array}$$

Energy conversion within Engine 5 leads to:

(39) $$J_{5,1}^{\alpha }Y_{5,1}^{\alpha }+J_{5,2}^{\alpha }Y_{2}^{\alpha }=-\left(J_{5,3}^{\beta }Y_{3}^{\beta }+J_{5,4}^{\beta }Y_{5,4}^{\beta }\right).$$

Considering an energy conversion direction from extensity α to β, the above model can be seen as a series of two smaller setups. The first one converts energy from extensity α to power output $P_{\mathrm{out}}^{\alpha }$, and the second one converts energy from its energy input, which is the output of the first one, $P_{\mathrm{in}}^{\beta }=P_{\mathrm{out}}^{\alpha }$, to extensity β. Now, if these smaller setups have the efficiencies $\eta_{G}^{\alpha }$ and $\eta_{G}^{\beta }$, respectively, one would expect them to multiply for the overall setup.

That this applies to the described engine setup can be shown by defining the loss fluxes analogous to Equations (28) and (32) as:

(40) $$\begin{array}{cc} J_{6}^{\alpha } & =J_{1}^{\alpha }\left(\eta_{G}^{\alpha }\frac{Y_{1}^{\alpha }}{Y_{5,1}^{\alpha }-Y_{5,2}^{\alpha }}-1\right), \end{array}$$

(41) $$\begin{array}{cc} J_{7}^{\beta } & =J_{4}^{\beta }\left(\frac{1}{\eta_{G}^{\beta }}\frac{Y_{3}^{\beta }}{Y_{5,3}^{\beta }-Y_{5,4}^{\beta }}-1\right). \end{array}$$

The power input into the engine setup is the (negative) energy flux leaving Reservoir 1:

(42) $$P_{\mathrm{in}}=-J_{1}^{\beta }Y_{1}^{\beta }$$

and the power output can then be expressed and rearranged as:

(43) $$\begin{array}{cc} P_{\mathrm{out}}\; & =J_{3}^{\beta }Y_{3}^{\beta }+J_{4}^{\beta }Y_{4}^{\beta } \end{array}$$

(44) $$\begin{array}{cc} \; & =-J_{1}^{\alpha }Y_{1}^{\alpha }\eta_{G}^{\beta }\eta_{G}^{\alpha }, \end{array}$$

where we used Equations (35) to (41). Therefore, the resultant engine efficiency is given by:

(45) $$\eta =\frac{P_{\mathrm{out}}}{P_{\mathrm{in}}}=\eta_{G}^{\alpha }\eta_{G}^{\beta }.$$

Such a multiplicative decomposition of the overall efficiency of an engine is by no means uncommon. Examples of these are thermal or volumetric efficiencies covering heat losses and leakages, respectively, which occur in combination with a mechanical efficiency that generally covers friction occurring in the system. For the sake of simplicity, a black box model can also be used for the above described rather complex engine setup with two extensity fluxes. Figure 5b illustrates this model where the reservoirs and the engine with given efficiency are reversibly connected. The entropy generated and transferred to Reservoir 8 can be written as:

(46) $$\sigma =J_{8}^{S}=J_{8,1}^{S}+J_{8,2}^{S}=J_{5,1}^{\alpha }\frac{Y_{1}^{\alpha }-Y_{5,1}^{\alpha }}{T_{8}}+J_{6}^{\alpha }\frac{Y_{1}^{\alpha }-Y_{6}^{\alpha }}{T_{8}}+J_{5,4}^{\beta }\frac{Y_{4}^{\alpha }-Y_{5,4}^{\alpha }}{T_{8}}+J_{7}^{\beta }\frac{Y_{4}^{\alpha }-Y_{7}^{\alpha }}{T_{8}}.$$

## 7. Example: AC Motor

We now want to give an example on how to model an engine with given efficiency. The engine that serves as an example is an AC (Alternating Current) motor described and tested in [50]. For this engine, input and output powers, efficiency and occurring losses are measured or calculated and presented as functions of engine speed *n* and output torque τ. The losses are divided into, and separately given as, copper loss, which is energy loss due to electric currents in windings, and iron and mechanical losses due to friction, magnetic hysteresis, and electromagnetic induction. This separation corresponds to the occurring energy flows which are carried by the electric current and the torque, respectively.

**Figure 6:**
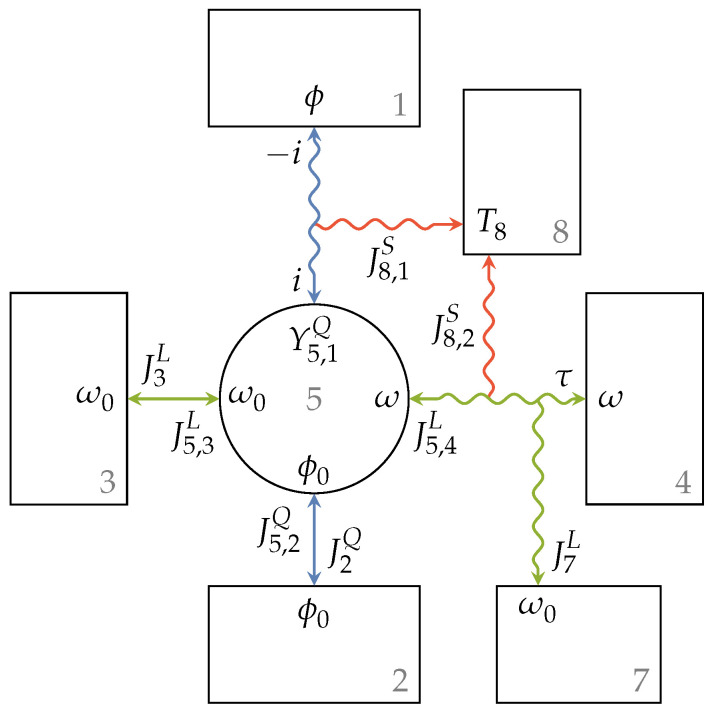
Full endoreversible engine setup used to model an AC motor. Compared to Figure 5a, we now have charge and angular momentum fluxes so that α=Q and β=L, respectively, and there is no loss flux towards Reservoir 6.

Figure 6 shows the full endoreversible model of the named AC motor with efficiency η(ω,τ), where ω=2πn is the angular velocity of the engine. The introduced dissipative engine setup is used to describe the energy transfer from an electric current *i* to a torque output τ, or in other words, from a charge flux $J_{1}^{Q}$ to an angular momentum flux $J_{4}^{L}$, respectively. The involved extensities are thus charge (α=Q) and angular momentum (β=L). The electric current flows from a high electric potential $\phi =Y_{1}^{Q}$ to a low electric potential $\phi_{0}=Y_{2}^{Q}$ causing a torque flow from a low angular velocity $\omega_{0}=Y_{3}^{L}$ to a high angular velocity $\omega =Y_{4}^{L}$. In the case of the AC motor, there is no loss flux of charge, but a voltage drop from ϕ to $Y_{5,1}^{Q}$ occurs. On the other hand, torque is transferred with the angular momentum ω, and a loss flux $J_{7}^{L}$ towards Reservoir 7 occurs. The generated entropy is transferred to Reservoir 8, which is a heat bath with temperature $T_{8}$. The lower intensities $\omega_{0}$ and $\phi_{0}$ are set to zero, which is a reasonable assumption for the case that the engine is mounted to the ground or a vehicle’s chassis, for instance.

From the extensity balance equation, we know that:

(47) $$\begin{array}{cc} J_{3}^{L} & =-J_{5,3}^{L}=J_{5,4}^{L}=-\tau -J_{4}^{L}, \end{array}$$

(48) $$\begin{array}{cc} J_{2}^{Q} & =-J_{5,2}^{Q}=i. \end{array}$$

The input and output power of this endoreversible engine setup are given as:

(49) $$\begin{array}{cc} P_{\mathrm{in}} & =-J_{1}^{Q}Y_{1}^{Q}=i\phi , \end{array}$$

(50) $$\begin{array}{cc} P_{\mathrm{out}} & =J_{4}^{L}Y_{4}^{L}=\tau \omega , \end{array}$$

respectively. The entropy production rates due to copper, as well as iron and mechanical losses can be written as:

(51) $$\begin{array}{cc} J_{8,1}^{S} & =\frac{I_{8,1}}{T_{8}}=\frac{i\left(\phi -Y_{5,1}^{Q}\right)}{T_{8}}=\sigma_{\mathrm{Cu}}, \end{array}$$

(52) $$\begin{array}{cc} J_{8,2}^{S} & =\frac{I_{8,2}}{T_{8}}=\frac{\tau_{\mathrm{loss}}\omega }{T_{8}}=\sigma_{\mathrm{FeMec}}, \end{array}$$

and the overall efficiency of the AC motor, which is a function of torque output and engine speed, shall be:

(53) $$\eta \left(\omega ,\tau \right)=\frac{P_{\mathrm{out}}}{P_{\mathrm{in}}}.$$

**Figure 7:**
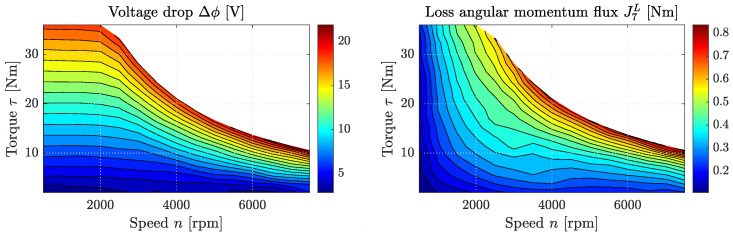
Loss charge flux $J_{6}^{Q}$ (left) and loss angular momentum flux $J_{7}^{L}$ (right) over engine speed *n* and output torque τ of the AC (Alternating Current) motor investigated in [50] using the introduced dissipated engine model.

In our example, $P_{\mathrm{Cu}}=I_{8,1}$ and $P_{\mathrm{FeMec}}=I_{8,2}$ are given as functions of the torque output and the engine speed. Using the above equations, we can thus easily determine the unknown voltage drop $\Delta \phi =\phi -\phi_{i}$, as well as the occurring loss flux of torque $J_{7}^{L}$ as shown in Figure 7. An interesting effect that can be seen here is that Δϕ mainly increases with output torque and has the highest values at around 2000 rpm, while $J_{7}^{L}$ seems to increase with the product of torque output and engine speed.

**Figure 8:**
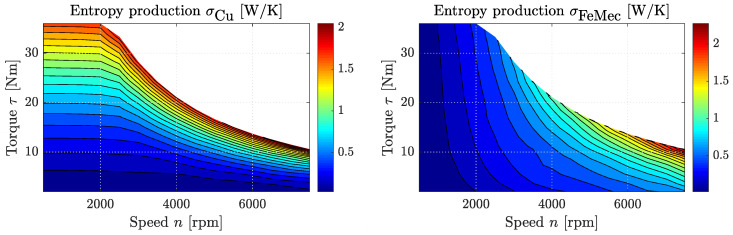
Entropy generation rates due to copper losses $\sigma_{\mathrm{Cu}}$ (left) and iron and mechanical losses $\sigma_{\mathrm{FeMec}}$ (right) over engine speed *n* and output torque τ of the AC motor investigated in [50] using the introduced dissipated engine model.

With temperature $T_{8}$ of Reservoir 8, which typically represents the environment at ambient temperature, we are also able to calculate the entropy production rate at every operation point of the engine. The assumption here is that both copper and iron and mechanical losses are converted into heat, which is ultimately released to the environment. Using Equation (46), the entropy fluxes and thus the entropy production rates can be calculated where $J_{8,1}^{S}=\sigma_{\mathrm{Cu}}$ and $J_{8,2}^{S}=\sigma_{\mathrm{FeMec}}$ are the entropy production rates due to copper and iron and mechanical losses, respectively. Figure 8 shows both of these rates over engine speed *n* and torque output τ. Here, it is interesting to see that both $\sigma_{\mathrm{Cu}}$ and $\sigma_{\mathrm{FeMec}}$ are of the same order of magnitude. Further, $\sigma_{\mathrm{Cu}}$ increases almost linearly when τ is increased from zero to its maximum value for given *n*, and $\sigma_{\mathrm{FeMec}}$ increases mainly with engine speed, while torque output has a growing influence at higher values of *n*.

## 8. Summary

Within the framework of endoreversible thermodynamics, we introduced a new, more general interaction type, which is not solely irreversible due to different intensities at the contact points of the two connected subsystems, but also due to the loss of the transferred extensity. That loss flux within the interaction usually enters a third subsystem at a lower intensity than those of the two main subsystems. Thus, a residual energy flux remains, which together with a generated entropy flux enters a further entropy contact point as heat. This leaky interaction provides a very useful extension to endoreversible modeling, especially when it comes to non-heat engines.

Using this new interaction type, we modeled a dissipative engine setup particularly suited for modeling and incorporating engines with given efficiency in large composite systems. The loss fluxes to achieve the desired efficiency for either operation directions were derived, and the entropy production of the setup was given. This was done for both a simplified model with unspecified power output or input and for a comprehensive model regarding two extensity fluxes. Since this model can also be viewed as a kind of black box model for engines with given efficiencies, it is especially suitable for engineering applications.

We demonstrated the modeling of an engine with given efficiency using data of an AC motor and the introduced dissipative engine setup. With the derived equations, we were able to calculate the occurring voltage drop of the electric current and the loss flux of the angular momentum transfer. The entropy production rates for both copper and iron and mechanical losses could also be displayed for the entire working range of the AC motor. The dissipative engine setup, as well as the more general leaky interaction, thus prove to be a useful and effective tool to incorporate engines with given efficiency into an endoreversible description while being consistent with the endoreversible formalism and conserving given information.
